# Sensing danger in the islet: the roles of pattern recognition receptors in β cells and type 1 diabetes

**DOI:** 10.3389/fimmu.2025.1677177

**Published:** 2025-09-03

**Authors:** Lixiang Tong, Yiting Tu, Shoujun Huang, Peilin Zheng

**Affiliations:** ^1^ Department of General Medicine, People’s Hospital of Longhua, Shenzhen, China; ^2^ School of Life Sciences, Anhui Agricultural University, Hefei, China; ^3^ Department of Neurology, The Fourth People's Hospital of Shenzhen (Shenzhen Samii Medical Center), Shenzhen, China

**Keywords:** pattern recognition receptors, innate immunity, pancreatic β cells, type 1 diabetes, autoimmunity

## Abstract

Type 1 diabetes (T1D) is an organ-specific autoimmune disease characterized by the immune-mediated destruction of pancreatic β cells, leading to absolute insulin deficiency and chronic hyperglycemia. Traditionally, the onset of T1D has been attributed to the interplay of genetic predisposition and environmental factors that disrupt immune tolerance. However, growing evidence suggests that β cells are not merely passive targets of immune attack. Instead, under conditions of inflammatory and metabolic stress, β cells actively participate in immune modulation by upregulating various immunologically relevant molecules, particularly pattern recognition receptors (PRRs). These innate immune sensors enable β cells to detect danger-associated signals and modulate local immune responses, thereby influencing their survival and immunogenicity. In this review, we summarize current knowledge about the expression profiles and immunoregulatory roles of PRRs in pancreatic β cells and explore their potential contributions to T1D pathogenesis. A deeper understanding of PRR-mediated signaling in β cells may provide novel insights into the immunopathology of T1D and reveal promising targets for therapeutic intervention.

## 1 Introduction

Type 1 diabetes (T1D) is a paradigmatic organ-specific autoimmune disease characterized by the immune system’s aberrant recognition and destruction of pancreatic β cells, resulting in progressive β-cell loss and absolute insulin deficiency (1, 2). Although T1D has a lower incidence than type 2 diabetes, it typically manifests at a younger age, progresses more rapidly, and has shown a steady global increase in incidence in recent decades, particularly among children and adolescents (3, 4).

The pathogenesis of T1D is multifactorial, driven by the interplay of genetic susceptibility (e.g., HLA-DR3-DQ2 or HLA-DR4-DQ8 genotype), environmental triggers (such as viral infections and gut microbiota dysbiosis), and both innate and adaptive immune responses (5–7). Growing evidence indicates that autoimmunity in T1D frequently precedes clinical onset and is primarily initiated by innate immune mechanisms. Innate immune cells, including B-1a cells, neutrophils, and plasmacytoid dendritic cells, sequentially contribute to the early phase of disease onset. By recognizing pathogen-associated molecular patterns (PAMPs) and damage-associated molecular patterns (DAMPs) released from injured or infected cells, the innate immune system triggers local inflammatory responses, creating a proinflammatory milieu that primes the adaptive immune system in mice (8, 9). During this process, damaged pancreatic β cells release autoantigens, such as insulin and glutamate decarboxylase (GAD), which are subsequently processed and presented by antigen-presenting cells (APCs) via major histocompatibility complex (MHC) molecules (10). This antigen presentation triggers the activation of autoreactive T cells and initiates a classical immune cascade (11). As the disease progresses, adaptive immune responses mediate the selective destruction of β cells, the pathological hallmark of T1D. CD8^+^ cytotoxic T lymphocytes are recognized as the principal effectors in β-cell destruction, whereas the roles of CD4^+^ helper T cells in the human islet remain less well defined, although they are thought to support CD8^+^ T-cell cytotoxicity and promote B-cell responses (12).

T1D has traditionally been viewed as an autoimmune disease driven by immune system dysfunction. Based on these findings, both antigen-specific and non-antigen-specific immunotherapies, such as oral insulin administration and Bacillus Calmette–Guérin vaccine immunization, have been explored to induce immune tolerance. However, these interventions have not yet achieved satisfactory clinical efficacy (13–16). To date, the only immunotherapy approved by the U.S. Food and Drug Administration for T1D prevention is the CD3 monoclonal antibody teplizumab, which can delay disease onset by approximately two years in high-risk individuals. Nonetheless, its long-term ability to halt disease progression remains uncertain (17). Collectively, these observations suggest that immune dysregulation alone may not fully explain the complex pathogenesis of T1D.

In recent years, increasing attention has been directed toward the active role of pancreatic β cells in the pathogenesis of T1D. Traditionally viewed as passive targets of immune attack, β cells are now recognized as active participants in immune regulation, particularly under inflammatory or stress conditions. Emerging evidence indicates that β cells can upregulate a range of innate immunity-related molecules, most notably pattern recognition receptors (PRRs), thereby acquiring immune-sensing capabilities (18). PRRs, including Toll-like receptors (TLRs), C-type lectin receptors (CLRs), NOD-like receptors (NLRs), RIG-I-like receptors (RLRs), and AIM2-like receptors (ALRs), trigger the expression of proinflammatory cytokines and/or type I interferons upon activation. In addition, they regulate non-transcriptional processes such as phagocytosis, autophagy, cytokine maturation, and programmed cell death, orchestrating the crosstalk between innate and adaptive immunity (19–21). The expression of multiple PRRs has been confirmed in β cells from both animal models and human pancreatic tissue (**Table 1**). Activation of these receptors elicits antiviral and inflammatory responses that may confer protective effects in certain contexts but can also enhance β-cell immunogenicity, potentially initiating or amplifying autoimmune responses under permissive conditions (22–25). These findings underscore the importance of investigating T1D pathogenesis through the lens of β-cell-intrinsic immunity and PRR-mediated danger sensing, which may yield novel insights into disease initiation and progression.

**Table 1:** Overview of β cell-associated PRRs implicated in type 1 diabetes.

Sensing category	PRR	Location	PAMPs	DAMPs
Bacterial structural ligands	TLR2	Plasma membrane	Lipoprotein, PGN	HSPs, HMGB1, OxLDL
	TLR4	Plasma membrane	LPS	HSPs, HMGB1, OxLDL, hyaluronan, β-defensins
	ALPK1	Cytosol	ADP-heptose	ND
Viral RNA	TLR3	Endosome	dsRNA (~40–50 bp)	Mitochondrial RNA
	MDA5	Cytosol	long dsRNA (>1 kb)	Mitochondrial dsRNA
	RIG-I	Cytosol	Short dsRNA (<1 kb), uncapped 5′ppp ssRNA	5’-Triphosphate self-RNA, Mitochondrial dsRNA
Cytosolic DNA	AIM2	Cytosol	Exogenous dsDNA	Host dsDNA
	TLR9	Endosome	Unmethylated CpG DNA	Mitochondrial DNA, nuclear self-DNA
	cGAS	Cytosol	Viral or bacterial DNA	Mitochondrial DNA, leaked genomic DNA
Cellular stress	NLRC5	Cytosol, Nucleus	ND	ND
	NLRP3	Cytosol	Various PAMPs	ATP, ROS, K^+^ efflux, mitochondrial DNA
	RAGE	Plasma membrane	ND	AGEs, HMGB1, S100 family proteins, amyloid-β peptides

PRR, pattern recognition receptor; PAMP, pathogen-associated molecular pattern; DAMP, damage-associated molecular pattern; TLR, Toll-like receptor; ALPK1, alpha kinase 1; MDA5, melanoma differentiation-associated protein 5; RIG-I, retinoic acid–inducible gene I; AIM2, absent in melanoma 2; NLRC5, NOD-like receptor family caspase recruitment domain–containing 5; NLRP3, NOD-, LRR-, and pyrin domain–containing protein 3; PGN, peptidoglycan; LPS, lipopolysaccharide; HMGB1, high-mobility group box 1; HSP, heat shock protein; ROS, reactive oxygen species; dsRNA, double-stranded RNA; ssRNA, single-stranded RNA; CpG-DNA, cytosine-phosphate-guanine DNA; ATP, adenosine triphosphate; cGAS, cyclic GMP-AMP Synthase; RAGE, receptor for advanced glycation endproducts; AGEs, advanced glycation end products; ND, not determined.

In this review, we systematically summarize the expression profiles of PRRs in pancreatic β cells, examine how their activation modulates β-cell function and contributes to T1D pathogenesis, and highlight potential implications for future mechanistic studies and therapeutic interventions.

## 2 Sensing bacterial danger: the role of TLR2, TLR4, and ALPK1 in β cells

**Figure 1:**
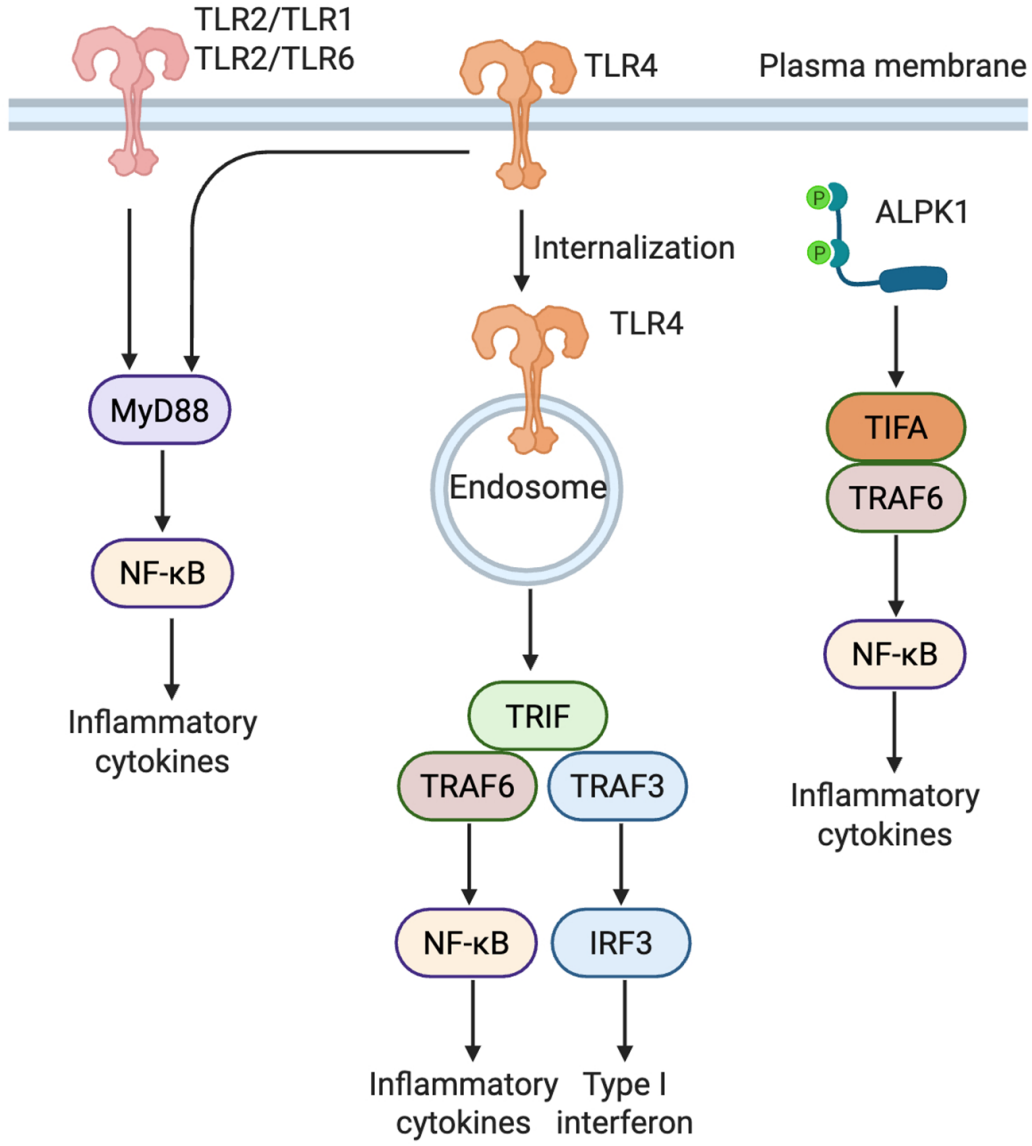
Distinct signaling pathways of TLR2, TLR4, and ALPK1 in β cells. TLR2 forms heterodimers with either TLR1 or TLR6 to recognize bacterial lipoproteins, triggering a MyD88-dependent NF-κB pathway that induces proinflammatory cytokines. TLR4 activates two distinct pathways: a MyD88-dependent response at the plasma membrane and a TRIF-dependent response following endocytosis, leading to activation of both NF-κB and IRF3 (Created in BioRender. Zheng, P (2025). https://BioRender.com/vnzzil2). ALPK1, upon detecting ADP-heptose, activates NF-κB through the TIFA-TRAF6 axis, independently of TLRs.TLR, Toll-like receptor; ALPK1, alpha kinase 1; MyD88, myeloid differentiation primary response 88; TRIF, TIR-domain-containing adapter-inducing interferon-β; TIFA, TRAF-interacting protein with FHA domain; TRAF, TNF receptor-associated factor; NF-κB, nuclear factor kappa-light-chain-enhancer of activated B cells; IRF3, interferon regulatory factor 3.

β cells express a variety of pattern recognition receptors that detect bacterial danger signals. Toll-like receptors, especially TLR2 and TLR4, are the most extensively studied and mediate recognition of microbial lipoproteins and lipopolysaccharides (LPS) at the cell surface. In parallel, alpha kinase 1 (ALPK1) has recently emerged as a cytosolic sensor of bacterial metabolites, providing a non-TLR pathway that complements TLR-mediated recognition (**Figure 1**).

### 2.1 Overview of TLR-Mediated signaling

TLRs, a prominent class of PRRs, are essential for the innate immune system’s capacity to detect microbial components. Structurally, TLRs feature extracellular leucine-rich repeats (LRRs) for ligand recognition, a transmembrane region, and a cytoplasmic Toll/IL-1 receptor (TIR) domain for signal transduction. To date, ten TLRs (TLR1-TLR10) have been identified in humans and twelve (TLR1-TLR9 and TLR11-TLR13) in mice, with TLR1 through TLR9 being highly conserved across both species (26).

TLR signaling proceeds primarily through two canonical pathways: the MyD88 (myeloid differentiation primary response 88)-dependent pathway and the TRIF (TIR-domain-containing adapter-inducing interferon-β)-dependent pathway (27–29). Most TLRs, except TLR3, signal via the MyD88 pathway. Uniquely, TLR4 engages both the MyD88-dependent and TRIF-dependent pathways, the latter via the adaptor protein TRIF-related adaptor molecule (TRAM). This dual signaling capacity enables coordinated activation of nuclear factor kappa-light-chain-enhancer of activated B cells (NF-κB) and interferon regulatory factor 3 (IRF3), thereby inducing both proinflammatory and antiviral responses (27, 30). Beyond detecting exogenous PAMPs, TLRs also recognize endogenous DAMPs, playing a pivotal role in sterile inflammation. DAMPs are endogenous molecules released or exposed by stressed, injured, or necrotic cells, acting as internal danger signals and potent activators of innate immunity (31–33). Building upon these core signaling mechanisms, we next examine how TLR2 and TLR4 contribute to the recognition of bacterial and endogenous danger signals in pancreatic β cells.

### 2.2 TLR2

TLR2 typically forms heterodimers with TLR1 or TLR6 to recognize bacterial lipoproteins and peptidoglycan (PGN). In addition to sensing PAMPs, TLR2 also recognizes endogenous DAMPs, such as heat shock proteins (HSPs), high-mobility group box 1 (HMGB1), and oxidized low-density lipoprotein (OxLDL) (34, 35).

Multiple studies have confirmed the expression of TLR2 in pancreatic β cells and related cell lines across both murine and human systems. In mice, reverse transcription polymerase chain reaction (RT-PCR) has detected TLR2 mRNA in primary islets (36–38). Similarly, human β cells express TLR2 transcripts, with significantly elevated levels observed in individuals with type 2 diabetes, suggesting a conserved role for TLR2 in β-cell stress sensing and immune regulation (36, 38). Murine and human β-cell lines also show consistent TLR2 mRNA expression, further supporting its involvement in β-cell physiology and innate immune responses (36–38).

The functional consequences of TLR2 activation in β cells appear to be context-dependent. Stimulation with TLR2 ligands such as PGN induces the expression of proinflammatory cytokines, including Tumor Necrosis Factor (TNF)-α, and Interleukin (IL)-6, in mouse islets and suppresses insulin secretion under high-glucose conditions, without significantly affecting β-cell viability (37). In contrast, β cells derived from TLR2/4-deficient mice retain normal insulin secretory responses to glucose and KCl stimulation. Furthermore, exposure of mouse and human islets to the TLR2 agonist lipoteichoic acid (LTA) and TLR4 agonist LPS markedly reduces β-cell proliferation under hyperglycemic conditions (38).

TLR2 has also been implicated in the pathogenesis of T1D. It has been proposed that TLR2 senses DAMPs released during β-cell apoptosis, thereby activating APCs and priming autoreactive T cells (39). In the multiple low-dose streptozotocin (STZ) model, TLR2-deficient mice exhibit delayed disease onset and progression (40). Similarly, under specific pathogen-free conditions, TLR2 deficiency reduces diabetes incidence in non-obese diabetic (NOD) mice; however, this protective effect is abrogated under germ-free conditions, highlighting a microbiota-dependent modulation of TLR2-mediated diabetogenicity (41).

Although β cell-specific TLR2 knockout (KO) models are currently unavailable, Bernd Krüger and colleagues employed an ectopic islet transplantation approach to investigate the β cell-intrinsic role of TLR2. In STZ-induced diabetic mice, transplantation of untreated syngeneic islets under the kidney capsule restored normoglycemia. In contrast, islets pretreated with PGN (a TLR2 agonist) failed to reverse hyperglycemia, whereas PGN-treated TLR2^-^/^-^ islets successfully normalized blood glucose levels. These findings highlight a detrimental role for β-cell TLR2 activation in islet survival and function (37). Collectively, these studies indicate that in mice TLR2 not only mediates proinflammatory signaling in immune cells but also directly contributes to β-cell dysfunction and the pathogenesis of T1D through its expression and activation in pancreatic β cells.

### 2.3 TLR4

TLR4, the first functionally characterized member of the TLR family, primarily recognizes LPS from Gram-negative bacteria and various DAMPs, including HMGB1, HSPs, S100 proteins, oxLDL, hyaluronan, and β-defensins (42, 43). Unlike most TLRs, TLR4 signals through both the MyD88- and TRIF-dependent pathways. At the plasma membrane, it engages MD-2 and CD14 to activate NF-κB via MyD88, thereby promoting the production of proinflammatory cytokine and chemokines. Upon endocytosis, TLR4 recruits TRAM and TRIF, leading to IRF3 activation and induction of type I interferons (44). Through this dual signaling, TLR4 acts as a key regulator of innate immune and inflammatory responses.

Expression of TLR4 mRNA and protein has been detected in pancreatic β cells from both mice and humans, as well as in β-cell lines (36–38, 45, 46). TLR4 expression is inducible by LPS stimulation, suggesting transcriptional regulation under inflammatory conditions. In NOD mice, TLR4 levels are relatively low during the pre-diabetic stage (4–6 weeks) but increase significantly during early disease progression (10–14 weeks), implicating TLR4 in the pathogenesis of T1D. Although HMGB1 can bind several receptors, including TLR2, TLR4, TLR9, and the receptor for advanced glycation endproducts (RAGE), evidence from isolated NOD islets indicates that HMGB1 preferentially engages TLR4 on β-cell surfaces, suggesting a link between HMGB1-TLR4 interaction and β-cell dysfunction (46).

The functional role of TLR4 in β cells remains controversial. Many studies support a proinflammatory, deleterious role. In both human and murine β cells, LPS-induced TLR4 activation upregulates inflammatory cytokines and chemokines (e.g., CCL2, TNF-α, IL-6, IL-8 and CXCL10), reduces insulin content, and impairs β-cell viability (37, 45, 47). *In vivo*, syngeneic islet transplantation restores normoglycemia in STZ-induced diabetic mice, but LPS-pretreated islets fail to reverse hyperglycemia (47). Suppression of TLR4 expression by carbon monoxide improves islet graft survival in xenotransplantation models (48). In addition, TLR4 deficiency reduces MyD88 and IRAK-1 phosphorylation, inhibits NF-κB activation, attenuates cytokine production, and alleviates islet inflammation in STZ-induced T1D models (49). Conversely, some studies suggest that TLR4 deficiency may not consistently confer protection and, under certain conditions, may even accelerate diabetes development. In NOD mice lacking TLR4, diabetes onset is accelerated, potentially due to altered gut microbiota or impaired regulatory T cell (Treg) function, suggesting an immunoregulatory role for TLR4 in maintaining immune tolerance (41, 50, 51). Moreover, β cells from TLR4-deficient NOD mice exhibit preserved function when exposed to inflammatory cytokines (e.g., IFN-γ, TNF-α, and IL-1β) or nitric oxide donors (e.g., DETA-NO), indicating that TLR4 may not be essential for mediating β-cell cytotoxicity under these conditions (50).

In summary, TLR4 plays a complex and context-dependent role in T1D pathogenesis. While it clearly promotes inflammation and contributes to β-cell dysfunction under certain conditions, it may also exert immunomodulatory functions that support immune homeostasis. Further studies are needed to delineate the molecular mechanisms that govern TLR4 signaling in β cells and its dualistic role in diabetes development.

### 2.4 ALPK1

ALPK1 is a recently characterized cytosolic kinase of the α-kinase family, comprising a conserved C-terminal kinase domain and an N-terminal domain linked by a flexible region. It selectively senses adenosine diphosphate (ADP)-heptose, a conserved metabolite in LPS biosynthesis (52, 53). Upon ligand binding, ALPK1 phosphorylates the adaptor protein TRAF-interacting protein with FHA domain (TIFA), recruits TRAF6, and activates the NF-κB pathway, leading to proinflammatory cytokine production (54). Notably, gain-of-function mutations in ALPK1 (e.g., p.Thr237Met, p.Tyr254Cys) cause ROSAH syndrome, a rare autoinflammatory disorder, underscoring its critical role in innate immune signaling (55).

Expression of ALPK1 mRNA and protein has been detected in the murine pancreatic β-cell line MIN6 (23). Inflammatory cytokines, such as IFN-γ, TNF-α, and IL-1β, significantly upregulate ALPK1 expression, whereas STZ, varying glucose concentrations, or ADP-heptose alone do not alter its expression, suggesting ALPK1’s involvement in cytokine-mediated β-cell stress responses. Further studies show that ALPK1 activation alone does not induce β-cell apoptosis. However, in the presence of proinflammatory cytokines, ADP-heptose mediated ALPK1 activation markedly exacerbates β-cell death. This synergistic effect likely results from enhanced TIFA phosphorylation and subsequent activation of transforming growth factor-β-activated kinase 1 (TAK1). As a key upstream kinase in the NF-κB pathway, TAK1 promotes expression of TNF-α and Fas, as well as caspase-3 activation, ultimately amplifying apoptotic signaling (23).

Animal studies further underscore ALPK1’s role in β-cell vulnerability. In transgenic C57BL/6 mice with systemic ALPK1 overexpression, baseline blood glucose levels remain normal; however, following repeated low-dose STZ administration, these mice exhibit more severe hyperglycemia and reduced insulin levels compared to wild type (WT) controls, indicating heightened β-cell susceptibility to damage (56). In the autoimmune diabetes model of NOD mice, ALPK1 expression in β cells progressively increases under chronic inflammatory stress, correlating with β-cell dysfunction during disease progression (57). Collectively, these findings suggest that dysregulated ALPK1 expression or activation may contribute to inflammation-induced β-cell apoptosis and play a pathogenic role in T1D development.

## 3 Sensing viral danger: the role of TLR3, MDA5, and RIG-I in β cells

**Figure 2:**
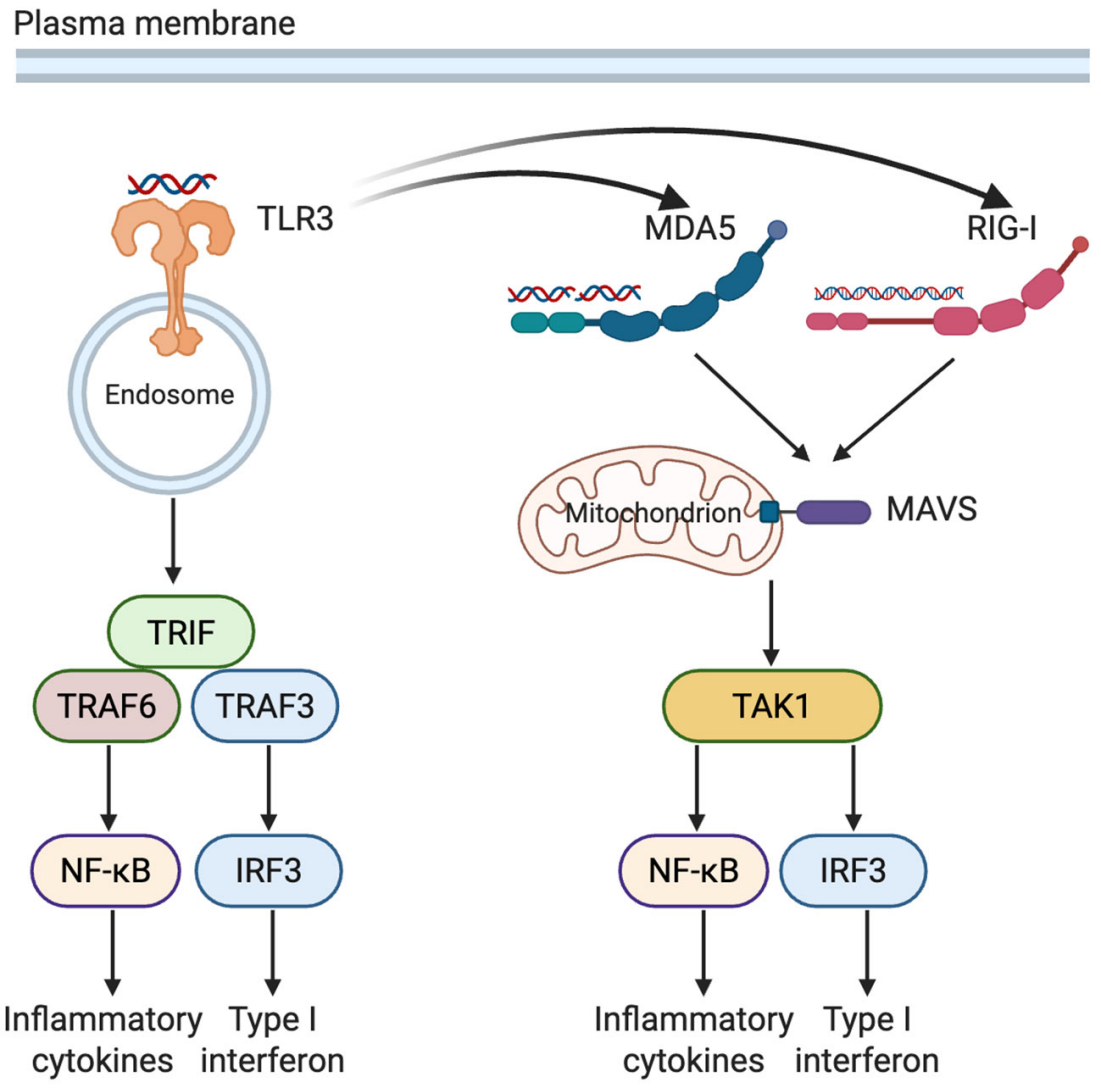
Complementary and synergistic recognition of viral RNA by TLR3, MDA5, and RIG-I in pancreatic β cells. TLR3, situated in endosomal membranes, detects extracellular or endocytosed dsRNA, triggering TRIF-dependent activation of NF-κB and IRF3. Within the cytosol, RIG-I and MDA5 recognize viral RNA species of varying lengths and structures, signaling through MAVS to activate the downstream kinase TAK1. Activation of TLR3 can upregulate expression of MDA5 and RIG-I. These pathways converge on NF-κB and IRF3, promoting the production of inflammatory cytokines as well as type I interferons (Created in BioRender. Zheng, P. (2025) https://BioRender.com/1xxxh1c). TLR3, Toll-like receptor 3; MDA5, melanoma differentiation-associated gene 5; RIG-I, retinoic acid-inducible gene I; TRIF, TIR-domain-containing adapter-inducing interferon-β; MAVS, mitochondrial antiviral-signaling protein; TAK1, transforming growth factor-β-activated kinase 1; TRAF3/6, TNF receptor-associated factor 3/6; NF-κB, nuclear factor kappa-light-chain-enhancer of activated B cells; IRF3, interferon regulatory factor 3; dsRNA, double-stranded RNA.

In addition to bacterial structural ligands, pancreatic β cells encounter viral pathogens, which are detected by a distinct group of intracellular and endosomal PRRs. This section reviews and discusses the roles and interactions of TLR3, melanoma differentiation-associated gene 5 (MDA5), and retinoic acid-inducible gene(RIG-I) in pancreatic β cells (**Figure 2**).

### 3.1 TLR3

TLR3, the only TLR family member that signals exclusively through the TRIF adaptor protein, resides in endosomal membranes and primarily recognizes double-stranded RNA (dsRNA) species longer than 40–50 base pairs, including viral replication intermediates and synthetic analogs like poly(I:C). TLR3 sensing, which is sequence-independent and driven by recognition of the RNA sugar-phosphate backbone, activates IRF3 and NF-κB signaling cascades. This leads to the induction of type I interferons (e.g., IFN-β), proinflammatory cytokines (e.g., IL-6, TNF-α) and chemokines such as CXCL10 and CCL5 (58, 59).

In pancreatic β cells, however, TLR3 exhibits limited functional activity compared to classical immune cells. Under basal conditions, TLR3 expression is minimal (60–62). Although exogenous poly(I:C) stimulation can upregulate TLR3 mRNA over 40-fold, it fails to induce corresponding increases in IFN-β transcripts in the INS832/13 β-cell line or in rat islet cells. Despite internalizing similar amounts of poly(I:C) as macrophages, β cells demonstrate defective activation of the TLR3-IFN signaling axis, possibly due to inefficient endosomal trafficking or impaired downstream signaling (63). Further studies highlight that the cellular localization of poly(I:C) dictates its impact on β cells. Extracellular poly(I:C) partially activates TLR3, leading to NF-κB activation and apoptosis, whereas intracellular delivery triggers robust β-cell death via a TLR3-independent mechanism involving protein kinase R (PKR) (63). This is consistent with findings that poly(I:C)-induced apoptosis persists in TLR3-deficient mice, indicating the involvement of non-TLR3 dsRNA-sensing pathways (64).

Functionally, TLR3 appears to play a pathogenic role in virus-induced T1D. Poly(I:C) administration exacerbates T1D in several mouse models (24, 64–66). In NOD mice, genetic deletion of TLR3 significantly reduces islet inflammation and lowers diabetes incidence following coxsackievirus B4 (CVB4) infection (67, 68). Beyond its immunologic function, TLR3 also modulates β-cell physiology. TLR3 deficiency enhances glucose- and K^+^-stimulated insulin secretion, an effect reversed by TLR3 activation (69). Moreover, TLR3 expression increases under metabolic stressors such as glucolipotoxicity, where it inhibits β-cell proliferation by suppressing cyclin D1/D2, inducing G1-phase arrest, and contributing to β-cell mass reduction (70).

### 3.2 MDA5 and RIG-I

MDA5 and RIG-I are cytosolic RNA sensors belonging to the RIG-I-like receptor (RLR) family, specialized in detecting viral dsRNA (71). Unlike TLR3, which resides in endosomal membranes and requires endocytosis for ligand delivery, RLRs directly sense cytoplasmic RNA. RIG-I preferentially recognizes short dsRNA (<1 kb) and uncapped 5’-triphosphate single-stranded RNA (ssRNA), while MDA5 primarily detects long dsRNA (>1 kb), particularly complex RNA networks generated during viral replication. Upon ligand recognition, both receptors signal through the mitochondrial antiviral signaling protein (MAVS), activating IRF3, IRF7, and NF-κB pathways, which induce type I interferons (IFN-α/β), chemokines (e.g., CCL2, CCL5 and CXCL10) and proinflammatory cytokines (72–74).

Both MDA5 and RIG-I are expressed in pancreatic endocrine cells, but with distinct expression patterns. MDA5 is significantly upregulated in both α and β cells from patients with recent-onset or fulminant T1D, with notably higher expression in α cells. In non-diabetic individuals, MDA5 expression is predominantly confined to α cells (75, 76). Interestingly, MDA5-positive but hormone-negative cell clusters have been observed in islets from recent-onset T1D patients, suggesting a possible involvement in β-cell dedifferentiation or regenerative processes (75). These findings imply that MDA5 may contribute to both β-cell destruction and inflammation-driven epigenetic remodeling. In contrast, RIG-I is minimally expressed in healthy or T1D control islets but is strongly induced in β cells from patients with enterovirus-associated fulminant T1D, indicating its role as an inducible stress sensor activated under acute viral infection (76).

Functionally, viral infection or stimulation with dsRNA analogs such as poly(I:C) robustly induces MDA5 and RIG-I expression, triggering type I interferon production, chemokine release and β-cell apoptosis. Knockdown of MDA5 or RIG-I in β cells attenuates poly(I:C)-induced interferon and inflammatory responses, yet fails to prevent apoptosis, suggesting that dsRNA-induced cell death involves additional pathways beyond RLR signaling (63, 77). In human β cells, IFN-α promotes human leukocyte antigen (HLA) class I upregulation together with inflammatory and ER stress responses, and synergizes with IL-1β to accelerate apoptosis (78). Importantly, it not only increases surface HLA-I levels but also remodels the β-cell immunopeptidome in human β cells, shifting peptide presentation toward HLA-B–restricted ligands. This bias facilitates activation of HLA-B–specific CD8^+^ T cells, consistent with the preferential HLA-B hyperexpression observed in islets from patients with T1D, where infiltrating cytotoxic T cells recognizing HLA-B–restricted granule peptides have been detected (79).

The role of MDA5 in T1D has been further explored in NOD mice using two genetic models targeting *Ifih1*, the gene encoding MDA5: a complete KO and a helicase domain 1 in-frame deletion (ΔHel1) that impairs ATPase activity. The ΔHel1 mutation delayed both spontaneous and coxsackievirus B3 (CVB3)-accelerated T1D onset, accompanied by reduced type I interferon levels and decreased infiltration of proinflammatory immune cells. In contrast, complete MDA5 deficiency did not confer protection and instead increased T1D incidence, particularly in males. These findings suggest a dual role for MDA5 in T1D pathogenesis: while partial loss-of-function may mitigate autoimmune activation and delay disease progression, complete loss may impair immune homeostasis and predispose to disease onset (80).

### 3.3 Cooperative viral RNA sensing by TLR3, MDA5, and RIG-I in β cells

TLR3, MDA5, and RIG-I exhibit complementary mechanisms in the detection of viral RNA. TLR3, localized to endosomal membranes, senses exogenous dsRNA internalized via endocytosis, making it particularly effective during the early stages of viral infection. In contrast, the cytoplasmic sensors MDA5 and RIG-I detect intracellular viral RNA generated during replication. MDA5 preferentially recognizes long dsRNA (>1 kb), whereas RIG-I detects short dsRNA (<1 kb). Together, these receptors form a comprehensive antiviral surveillance network based on RNA length, structure, and subcellular localization.

TLR3 activation can also upregulate the expression of MDA5 and RIG-I via TRIF-mediated signaling, thereby amplifying cytoplasmic antiviral responses. For instance, in rhinovirus-infected epithelial cells, TLR3 stimulation enhances RIG-I and MDA5 transcription (81). In dengue virus infection models, all three receptors synergistically promote IFN-β production; knockdown of any one receptor significantly increases viral replication, highlighting their functional interdependence (82). A similar mechanism appears to operate in pancreatic β cells, where poly(I:C) fails to induce MDA5 and RIG-I expression in TLR3-deficient islets (83).

In the context of T1D, activation of TLR3, MDA5, and RIG-I in β cells exerts both protective and pathogenic effects. During the initial phases of viral infection, these sensors trigger type I interferon production and antiviral protein expression, limiting viral replication within β cells. For example, in recent-onset T1D patient islets, elevated MDA5 expression correlates with Coxsackievirus capsid protein VP1 and type I IFN markers, suggesting a role in viral containment (84). *In vitro*, activation of these receptors in human islets elicits IFN responses that suppress CVB replication and help preserve β-cell function (85). However, this antiviral signaling can also amplify local inflammation. Activation of any one sensor can induce the expression of others, creating a feed-forward inflammatory loop (83). In epithelial cells and macrophages, pre-exposure to inflammatory stimuli exacerbates responses to secondary viral insults, such as SARS-CoV-2, underscoring the impact of immune priming (86). In T1D, persistent production of type I IFNs may contribute to a localized “interferonopathy”, disrupting Treg cell function and fostering autoimmune progression (87). Thus, while TLR3, MDA5, and RIG-I play crucial roles in β-cell antiviral defense, their sustained or dysregulated activation may inadvertently initiate or exacerbate autoimmune damage in T1D.

## 4 Sensing DNA: the role of AIM2, cGAS and TLR9 in β cells

Following the recognition of viral RNA, the sensing of double-stranded DNA (dsDNA) represents another key layer of innate immune defense in β cells, mediated primarily by absent in melanoma 2 (AIM2) and cyclic GMP-AMP Synthase (cGAS) in the cytosol, together with TLR9 in endosomal compartments.

### 4.1 AIM 2

AIM2 is a member of ALR family that detect dsDNA of host or microbial origin. Upon ligand recognition, AIM2 forms the AIM2 inflammasome complex, activating caspase-1 and promoting the maturation and secretion of IL-1β and IL-18, as well as inducing pyroptotic cell death (88, 89). While AIM2 is predominantly expressed in hematopoietic cells, recent studies have detected low basal expression in healthy human pancreatic islets, with marked upregulation observed in the pancreatic tissue of individuals with T1D. In contrast, AIM2 expression in peripheral blood mononuclear cells remains unchanged, suggesting a tissue-specific role in T1D-associated islet inflammation (90).

Interestingly, AIM2 may exert a protective effect in T1D. In STZ-induced T1D mouse model, AIM2-deficient animals exhibit increased disease susceptibility, characterized by heightened islet inflammation, elevated blood glucose levels, and reduced insulin secretion (91). This protective effect has been attributed to AIM2’s role in preserving intestinal homeostasis, limiting microbial translocation to pancreatic-draining lymph nodes, and suppressing immune responses directed against β cells. Supporting this notion, islet transplantation studies showed that while WT islets restored normoglycemia in STZ-treated recipient mice, AIM2^-^/^-^ islets failed to do so and displayed elevated levels of p202—a negative regulator of type I and type II interferon signaling (92). These findings suggest that AIM2 may confer protection by restricting p202-mediated immunosuppressive signaling pathways that contribute to islet dysfunction.

In summary, AIM2 is basally expressed in pancreatic tissue and upregulated in T1D, implicating its involvement in local immune regulation. By maintaining gut-pancreas immune homeostasis and modulating interferon pathways, AIM2 may serve a protective role in islet preservation. However, the lack of β-cell specific AIM2 conditional knockout models limits mechanistic understanding of its cell-intrinsic functions in islet immunity, highlighting the need for further investigation.

### 4.2 cGAS

cGAS acts as a cytosolic DNA sensor that detects double-stranded DNA (dsDNA) of microbial or endogenous origin. DNA binding induces a conformational rearrangement in cGAS, activating its enzymatic function to catalyze the production of the cyclic dinucleotide second messenger cyclic GMP–AMP (cGAMP) from ATP and GTP. cGAMP directly engages stimulator of interferon genes (STING), an adaptor protein anchored in the endoplasmic reticulum membrane, triggering its dimerization and relocalization to perinuclear compartments. There, STING recruits and activates TANK-binding kinase 1 (TBK1), which phosphorylates IRF3, leading to its nuclear translocation and induction of type I interferons. Concurrently, STING signaling activates the NF-κB pathway, driving the expression of proinflammatory cytokines and chemokines such as CXCL10 and CCL5 (93, 94). Collectively, the cGAS–STING pathway represents an important signaling axis that couples cytosolic DNA sensing to both antiviral defense and sterile inflammatory responses.

cGAS is expressed in human and mouse pancreatic β cells as well as in β-cell lines, and its expression is regulated by hyperglycemic conditions. In diabetic mouse islets and MIN6 cells exposed to high glucose, cGAS expression is markedly upregulated (95). Functionally, cGAS exerts a negative regulatory role in β-cell proliferation. Both global and β-cell–specific deletion of cGAS enhance β-cell mass and improve glucose tolerance in mice, an effect likely mediated through a STING-independent mechanism that reduces the expression of the known β-cell proliferation inhibitor CCAAT/Enhancer-Binding Protein Beta (CEBPβ) (95). Thus, glucose-induced upregulation of cGAS may impair β-cell regenerative capacity and quality, thereby contributing to the onset and progression of diabetes. Metabolic stress and aging promote the release of mitochondrial DNA into the cytosol of β cells, thereby activating the cGAS–STING pathway and inducing a senescence-associated secretory phenotype (SASP)-like inflammatory state; this process can be alleviated by small-molecule STING inhibitors such as C176 (96).

In T1D, investigations of the cGAS–STING signaling axis remain relatively limited. Experimental evidence shows that treatment with a STING agonist in prediabetic NOD mice significantly delays disease onset and reduces incidence, potentially through enhanced indoleamine 2,3-dioxygenase (IDO) activity in both peripheral immune tissues and pancreas (97). Conversely, genetic deletion of STING in NOD mice accelerates diabetes development. NOD.STING^-^/^-^ mice exhibit increased numbers of autoreactive CD8^+^ T cells in peripheral lymphoid tissues, and splenocytes from these mice induce diabetes more rapidly upon transfer into irradiated NOD recipients (98). These findings suggest that STING deficiency enhances the pathogenicity of immune cells and accelerates disease progression. Notably, although STING plays a role in type I interferon production, the accelerated diabetes observed in STING-deficient mice appears independent of interferon induction and may instead reflect a role of STING in controlling autoreactive T cells. Further studies are warranted to fully elucidate the underlying mechanisms.

### 4.3 TLR9

TLR9, primarily localized to intracellular endosomal compartments, recognizes unmethylated CpG motifs present in bacterial and viral DNA, as well as, to a lesser extent, host-derived DNA released from damaged or dying cells (26). Upon ligand engagement, TLR9 signals through the MyD88 adaptor protein, activating NF-κB and IRF7 pathways and inducing the production of proinflammatory cytokines and type I interferons (IFN-α/β) (99).

Although widely expressed in immune cells, TLR9 is also present in pancreatic β cells. Functionally, TLR9 appears to negatively regulate β-cell development and function. In both NOD and C57BL/6 mouse strains, TLR9 deficiency is associated with increased β-cell mass, improved glucose tolerance, and enhanced insulin sensitivity (100).

In the context of T1D, genetic ablation or pharmacological inhibition of TLR9 (e.g., via chloroquine) protects NOD mice from disease onset (101, 102). Notably, B cell-specific deletion of TLR9 in NOD mice nearly abolishes T1D development and is accompanied by an expansion of IL-10-producing regulatory B cells (103). These findings underscore the immunomodulatory role of TLR9 in shaping autoimmune responses. However, its precise function within pancreatic β cells during T1D pathogenesis remains poorly defined and warrants further investigation.

## 5 Sensing cellular stress: the role of NLRs and RAGE in β cells

The human NLR family comprises approximately 22 members, while around 34 have been identified in mice. Structurally, NLRs share a conserved tripartite architecture: an N-terminal signaling domain that mediates protein-protein interactions, a central nucleotide-binding oligomerization domain responsible for ATP-dependent self-activation, and a C-terminal LRR domain involved in ligand sensing (104, 105). Based on their N-terminal motifs, NLRs are categorized into several subfamilies, including NLRA, NLRB, NLRC, NLRP, and NLRX (106, 107). NLRs are pivotal in innate immune sensing, antigen presentation, and inflammation. In pancreatic β cells, NLRC5 (NOD-like receptor family caspase recruitment domain–containing 5) and NLRP3 (NOD-like receptor family pyrin domain-containing 3) are the most extensively studied members.

### 5.1 NLRC5

NLRC5 is a master regulator of MHC-I gene transcription (108, 109). Increasing evidence suggests that NLRC5 contributes to modulating the immune visibility of β cells. In a study analyzing human pancreatic islets from 23 donors, NLRC5 expression was detected in 16 individuals and found to be significantly upregulated in β cells from T1D patients compared to healthy controls. Functional studies demonstrated that NLRC5 knockdown in β cells attenuates IFN-α–induced MHC-I upregulation and decreases the transcription of multiple genes involved in antigen processing and presentation. In addition, loss of NLRC5 limited the generation of neoantigens arising from alternative splicing, cis-splicing, and post-translational modifications, thereby narrowing the β-cell immunopeptidome. This reduction in antigen diversity was functionally relevant, as it led to suppressed activation and cytotoxicity of autoreactive CD8^+^ T cells in co-culture assays. In contrast, intact NLRC5 expression amplified β-cell antigenicity under type I interferon stimulation, promoting enhanced HLA-I surface density and broadening the peptide repertoire presented to T cells. Together, these findings position NLRC5 as a pivotal transcriptional amplifier of IFN-driven immunogenic remodeling in pancreatic β cells (110).

### 5.2 NLRP3

NLRP3 is a key inflammasome component and central mediator of sterile inflammation in β cells (111, 112). It senses diverse pathogen- or damage-associated signals, including bacterial RNA, ATP, mitochondrial DNA, uric acid crystals, and advanced glycation end products (AGEs). Under stress conditions such as hypoxia or oxidative insult, NLRP3 expression is upregulated in β cells, triggering inflammasome assembly. This process activates caspase-1 through recruitment of apoptosis-associated speck-like protein containing a CARD (ASC) and pro-caspase-1, promoting the maturation and secretion of IL-1β and IL-18, which drive inflammatory responses and impair β-cell viability (113–118).

NLRP3 signaling has been strongly implicated in T1D pathogenesis. In STZ-induced diabetic mice, knockout of either NLRP3 or ASC reduces hyperglycemia and preserves β-cell viability (118). Similarly, NLRP3-deficient NOD mice are protected from spontaneous T1D onset. Mechanistically, NLRP3 deletion downregulates the expression of IRF-1-dependent chemokines (CCL5 and CXCL10) in islets and reduces their receptors (CCR5 and CXCR3) on T cells, thereby limiting pathogenic T cell migration into the islets (119). Moreover, transplantation of NLRP3-deficient islets into diabetic recipients improves graft function and glycemic control (120). Collectively, these findings highlight the critical role of NLRP3-mediated inflammation in β-cell injury and autoimmune progression in T1D.

### 5.3 RAGE

Unlike classical PRRs for PAMPs, RAGE primarily recognizes endogenous DAMPs, such as AGEs, HMGB1, members of the S100 protein family, and β-amyloid peptide (Aβ) (121). Upon activation, RAGE initiates multiple downstream signaling cascades, including the mitogen-activated protein kinase (MAPK), phosphoinositide 3-kinase/protein kinase B (PI3K/Akt), and Janus kinase/signal transducer and activator of transcription (JAK/STAT) pathways, and robustly activates NF-κB. NF-κB not only induces the transcription of pro-inflammatory mediators such as TNF-α, IL-1β, IL-6, and MCP-1, but also upregulates RAGE expression through a positive feedback loop, establishing a self-amplifying inflammatory circuit. In parallel, RAGE stimulates Nicotinamide Adenine Dinucleotide Phosphate Hydrogen (NADPH) oxidase activity, promoting the generation of reactive oxygen species (ROS) that exacerbate oxidative stress and cellular injury (122).

Importantly, RAGE does not act in isolation but displays substantial functional synergy with TLRs. For example, in bovine alveolar macrophages, inflammatory responses are synergistically amplified by combined exposure to high glucose with LPS or HMGB1 with LPS (123). Moreover, RAGE promotes the internalization of DNA or RNA into host endosomes, thereby enhancing NF-κB activation and increasing the sensitivity of ssRNA-sensing TLRs (124, 125). At the receptor expression level, RAGE and TLRs exhibit reciprocal regulation: inhibition of RAGE decreases TLR4 expression, whereas suppression of TLR4 reduces RAGE expression (123). Collectively, these findings demonstrate that RAGE functions not only as a DAMP receptor directly driving inflammation but also as a critical amplifier of inflammatory signaling through its crosstalk with TLRs.

RAGE is expressed in multiple cell types, including pancreatic β cells. Studies have demonstrated that toxic precursors of human islet amyloid polypeptide (IAPP) and glycated serum can induce upregulation of RAGE expression in both β-cell lines and primary islets (126, 127). In human diabetic pancreatic tissues, increased RAGE expression in β cells is closely associated with islet amyloid deposition. Functionally, RAGE selectively binds to toxic IAPP intermediates and subsequently activates NADPH oxidase and triggers inflammatory responses, which play critical roles in the protein toxicity associated with islet amyloid deposition (126, 128). *In vitro* and *ex vivo* studies have further confirmed that RAGE ligands, including S100 family proteins and HMGB1, induce oxidative stress and apoptosis in β-cell lines and isolated islets, effects that can be attenuated by antioxidants or NADPH oxidase inhibitors (129). Glycated serum similarly enhances β-cell apoptosis, whereas blockade of RAGE with neutralizing antibodies or RAGE knockdown effectively abrogates these deleterious effects, providing direct evidence for the involvement of RAGE in β-cell death (127). Moreover, AGEs reduce insulin secretion by suppressing the expression of the key transcription factor pancreatic and duodenal homeobox 1 (Pdx-1), while RAGE-blocking antibodies restore Pdx-1 expression and insulin mRNA levels in INS-1 cells (130).

The RAGE signaling pathway also plays a pivotal role in the pathogenesis of T1D. Short-term administration of soluble RAGE (sRAGE), functioning as a decoy receptor, effectively prevents or delays the onset of diabetes, likely through expansion of Tregs in islets, pancreatic lymph nodes, and spleen (131). In adoptive transfer models, sRAGE treatment similarly suppresses β-cell destruction mediated by splenocytes from diabetic mice; however, it fails to protect against diabetes induced by transfer of activated pathogenic BDC2.5 CD4^+^ T-cell clones. These findings suggest that RAGE/ligand interactions may contribute to the differentiation of T cells toward a mature pathogenic phenotype during later stages of diabetes progression (132). Taken together, current evidence indicates that RAGE contributes to T1D pathogenesis both by mediating β-cell intrinsic stress and by modulating immune responses.

## 6 Conclusion and perspectives

Recent research has increasingly focused on the role of pancreatic β cells, which serve as the primary targets in T1D, under inflammatory and infectious conditions. Emerging evidence reveal that β cells are not merely passive victims of autoimmune attack but actively contribute to disease pathogenesis by expressing various PRRs. Upon activation, these receptors trigger the production of proinflammatory cytokines and type I interferons, thereby modulating β-cell function, viability, and local immune responses.

Despite substantial progress, many mechanistic aspects of β cell-intrinsic PRR signaling in T1D remain unresolved. First, the temporal dynamics and regulatory mechanisms governing PRR expression and activity throughout different stages of disease progression are poorly understood. Advances in single-cell RNA sequencing (scRNA-seq), spatial transcriptomics, and other multi-omics technologies now offer unprecedented opportunities to dissect β-cell molecular profiles in both spatial and temporal dimensions. Future studies can harness these tools to systematically map the expression landscapes and regulatory networks of PRR families in β cells across the T1D continuum. Such efforts may illuminate how PRR-mediated stress responses evolve with disease and help identify stage-specific therapeutic windows.

Second, the functional consequences of PRR activation within β cells remain incompletely defined, partly due to the lack of β cell-specific PRR knockout models. Developing such tissue-restricted genetic tools will be essential for disentangling local β cell-intrinsic effects from systemic immune responses mediated by infiltrating leukocytes.

In addition, there is an urgent need for precision strategies to modulate PRR activity in a context- and cell-type-specific manner. Emerging studies on MDA5 illustrate the complexity of PRR function: while partial loss-of-function mutations can delay T1D onset, complete ablation may impair immune homeostasis and exacerbate disease (83). These findings underscore the importance of nuanced, dose- and time-sensitive modulation of PRRs, rather than broad-spectrum inhibition, and support the development of selective small-molecule inhibitors or biologics tailored for β-cell protection.

Translationally, the priority is to convert β-cell PRR circuitry into stage-specific, tissue-selective interventions that preserve endogenous insulin secretion. Building on the findings summarized above, it is reasonable to hypothesize that the clinical relevance of β-cell PRRs is most pronounced in the early stages of T1D, when β cells act as danger sensors participating in local inflammatory cascades. Early T1D, defined as Stage 1 (multiple islet autoantibodies with normoglycemia) and Stage 2 (multiple autoantibodies with dysglycemia but without clinical symptoms), is characterized by β-cell stress and early immune activation (133). In this context, partial dampening of PRR signaling could reduce interferon-driven stress and chemokine release, thereby delaying immune cell infiltration and preserving β-cell resilience. Once autoimmunity becomes T cell–dominated in new-onset or established disease, PRR-based strategies are unlikely to serve as stand-alone interventions but could still be explored as adjunctive, time-limited approaches—for example, peri-infection modulation to prevent further β-cell loss. Overall, the translational promise of targeting β-cell PRRs resides primarily in early, stage-specific interventions, consistent with the concept of β cells as active danger sensors in islet autoimmunity.

In summary, PRRs in pancreatic β cells constitute a dynamic innate immune surveillance network. Far from being passive targets, β cells actively shape immune responses and determine their own fate through PRR signaling. Depending on disease stage and microenvironmental context, PRRs may exert either protective or pathogenic effects, reflecting their dual and context-dependent roles. A deeper understanding of the temporal regulation, signaling integration, and downstream functional outcomes of β cell-intrinsic PRRs will be critical for identifying key regulatory nodes and actionable therapeutic targets—ultimately enabling earlier intervention and the development of precision therapies for T1D.
